# *Cryptosporidium* in countries of the Arab world: the past decade (2002–2011)

**DOI:** 10.3402/ljm.v7i0.19852

**Published:** 2012-11-27

**Authors:** Khalifa Sifaw Ghenghesh, Khaled Ghanghish, Hanan El-Mohammady, Ezzddin Franka

**Affiliations:** 1 Faculty of Medicine, University of Tripoli, Tripoli, Libya; 2 Faculty of Veterinary Medicine, University of Tripoli, Tripoli, Libya; 3 US Naval Medical Research Unit-3, Cairo, Egypt

**Keywords:** *Cryptosporidium*, cryptosporidiosis, diarrhea, children, foreign housekeepers, Arab countries

## Abstract

**Introduction:**

*Cryptosporidium* is the causative agent of cryptosporidiosis. The disease is self-limited in immunocompetent persons but potentially life-threatening in immunocompromised individuals.

**Methods:**

The data included in the present review were obtained mainly from a Highwire Press (including PubMed) search for the period 2002–2011.

**Results:**

Information on cryptosporidiosis is lacking in some Arab countries; however available data show prevalence rates of <1–43% (mean = 8.7%) of *Cryptosporidium* infection in diarrheic immunocompetent pediatrics and <1–82% (mean 41%) in immunocompromised patients (including children and adults). Infection rate with *Cryptosporidium* species among pediatrics in rural and semiurban areas was higher than in urban areas. *Cryptosporidium*-associated diarrhea occurs mainly in younger children and inversely correlates with age, being more prevalent in children aged 1 year or less, particularly in rural and suburban regions. Although most Arab countries are characterized by a hot summer and a mild winter, infection with *Cryptosporidium* appears to occur at a higher rate of incidence during the rainy months that are usually associated with the cold season of the year. Contact with animals and contaminated waters are the main modes of transmission of cryptosporidia. Reports of *C. hominis* from the region indicate that person-to-person transmission is also important. Foreign housekeepers in oil-rich countries may be a source of *Cryptosporidium*.

**Conclusion:**

*Cryptosporidium* species, mainly *C. parvum*, are important causes of diarrhea in countries of the Arab world, particularly in children. In addition to educational programs that promote personal, household, as well as food hygiene, improving water treatment processes and protection of treated waters from contamination should be implemented by the health and environmental authorities in each country. More studies employing molecular testing methods are needed in the future to provide data on circulating species/genospecies and subtypes and their modes of transmission in the community.

*Cryptosporidium* is the causative agent of cryptosporidiosis. The disease is self-limited in immunocompetent persons but potentially life-threatening in immunocompromised individuals (e.g. those with the acquired immunodeficiency syndrome [AIDS]). *Cryptosporidium* species are members of the family *Cryptosporidiidae* (1). There are more than 20 species in the genus *Cryptosporidium* that have been described on the basis of the animal hosts from which they were isolated, parasite morphology, host predilection, and infection site (2). However, five species/genotypes of *Cryptosporidium* are mostly associated with cryptosporidiosis cases in immunocompetent and immunocompromised individuals, including *C. hominis*, *C. meleagridis*, *C. felis*, *C. canis*, and *C. parvum*, with predominance of the latter organism (3, 4). *C. hominis*, as the species name indicates, infects mainly humans (*Homo sapiens*).

In some countries of the Arab world, *Cryptosporidium* species are shown to be an important cause of diarrhea in pediatrics and in immunocompromised patients (5–8). The aim of this minireview is to provide an overview, on the basis of data available in the literature between 2002 and 2011, with respect to prevalence, clinical symptoms, sources of infection, and treatment and prevention of cryptosporidiosis in the Arab countries.

The data included in the present review were obtained from a Highwire Press (including PubMed) search for the period 2002–2011 using the terms ‘*Cryptosporidium*+name of Arab country (e.g. Libya, Egypt, etc.)’, ‘cryptosporidiosis + name of Arab country’, and ‘intestinal parasites + name of Arab country’. Supplementary data were also obtained from a Google search using the aforementioned terms.

Although information on cryptosporidiosis is lacking in some Arab countries, available data show prevalence rates of 1–43% (mean = 8.7%) of *Cryptosporidium* infection in diarrheic immunocompetent pediatrics (Supplemental table 1). Prevalence rates differ widely among countries and within the same country. For example, rates of 2% and 37% were reported from Jordan and 2% and 13% from Libya (Supplemental table 1). This may be due to differences in the number of patients examined, type of populations studied (e.g. urban or rural), location, and year of study. There are no studies examining the prevalence of cryptosporidia in adult populations. However, a study from Jordan reported *Cryptosporidium* species in 8.3% (15/180) of patients with diarrhea aged 12–84 years (mean = 48 years) and from none (0.0%) of 100 controls (8).

Methods used in the detection of the organism may also play a role in the reported differences of prevalence rates. The majority of studies examined single stool samples from humans and animals. The reported prevalence rates could have been higher if three specimens were examined as recommended (9). The use of molecular procedures may overcome this problem. Helmy et al. examined 50 single stool samples from patients with severe diarrhea (10). They detected *Cryptosporidium* in 22 (44%) samples by microscopy. When the same samples were examined by PCR using primers specific for DNA of *C. parvum* the organism was detected in 28 (56%) samples. Their study concluded that microscopy exhibited 78.5% sensitivity and 100% specificity compared with 100% specificity and sensitivity with PCR.

Most studies report the detection of the organism in diarrheic children but not in controls (8, 11, 12). However, Al-Braiken et al. reported *Cryptosporidium* in 9% (17/190) of asymptomatic children aged less than 5 years attending six (randomly selected) crèches in Jeddah, Saudi Arabia (13). Also, Al-Harthi examined the prevalence of intestinal parasites in asymptomatic school children aged 7 to 12 years from 13 primary schools in Makkah, Saudi Arabia (14). *Cryptosporidium* was detected in 4% of 589 stool samples examined and was the predominant pathogenic parasite in the population studied.

Reports also show that the infection rate with *Cryptosporidium* species among pediatrics in rural and semiurban areas is higher than in urban areas (11, 15–18). Others reported no significant difference between urban and rural prevalence rates (7). *Cryptosporidium*-associated diarrhea occurs mainly in younger children and inversely correlates with age, being more prevalent in children aged 1 year or less, particularly in rural and suburban regions (5, 6, 15, 19).

Although most Arab countries are characterized by a hot summer and a mild winter, infection with *Cryptosporidium* appears to occur at a higher rate of incidence during the rainy months that are usually associated with the cold season of the year (15, 17).

## Immunocompromised patients

During the 10-year period of the review, six studies (four from North Africa) examined immunocompromised patients, including children and adults, for *Cryptosporidiu*m infection (Supplemental table 2) with prevalence rates ranging from <1 to 82% (mean = 41%). Low prevalence rates of *Cryptosporidium* infection were observed among non-diarrheic, immunocompromised individuals. On the contrary, rates of infection higher than 40% were reported mainly among immunocompromised patients with diarrhea, particularly diarrheic HIV patients. This may be due to the advancement of the immune-suppression state in the latter patients. Studies indicate that gender plays no role in the infection rates of the organism among immunocompetent female and male children with diarrhea. However, contrary to other studies, Sanad and Al-Malki found that *Cryptosporidium* infection was significantly higher in immunocompromised males than in female patients, and the infection was lowest among children under 2 years of age and the highest among patients aged 16 to 40 years (20).

## Clinical symptoms

Common clinical findings associated with *Cryptosporidium* infection in immunocompetent pediatrics are diarrhea, fever, and vomiting; these symptoms usually resolve spontaneously (6, 21). Dehydration and severe symptoms, which include persistent diarrhea requiring hospitalization, have also been reported (5, 12).

Several investigators reported that prolonged disease and more severe clinical presentation were associated with coinfection of *Cryptosporidium* with other enteric pathogens (8, 11). Others found no significantly different clinical symptoms between children infected with *Cryptosporidium* alone or in combination with other enteric pathogens (6). Ben Abda et al. identified five cryptosporidiosis cases with major histocompatibility complex class II deficiency syndrome among 11 Tunisian children with pelvic inflammatory disease (PID) (22); chronic diarrhea with failure to thrive was observed in all five children. In their study using molecular procedures, two cases were identified as *C. hominis*, one as *C. meleagridis*, one as *C. parvum*, and one as *C. hominis*/*C. meleagridis* coinfection.

## Sources of *Cryptosporidium* infection

### Foreign housekeepers

The role of foreign workers from Asia and Africa as a source of intestinal parasitic (including *Cryptosporidium* species) infection in Arab countries has not been well examined. A study from Saudi Arabia found high rates of parasitic infection (including *Cryptosporidium* species) among young children of high-income families, with more than 70% of these families having foreign maids who are usually in direct contact with such children (14). A high intestinal parasite prevalence rate of 46.5% among female Asian housekeepers was reported previously from Saudi Arabia (23). This may indicate that members of families with foreign housekeepers, particularly those families with children and immunocompromised individuals, are at high risk of infection from parasitic agents including *Cryptosporidium* species. However, more studies are needed in Arab countries where foreign housekeepers are common (e.g. Gulf and other oil-rich countries) to confirm such observation.

### Animals

*Cryptosporidium* species are established zoonotic enteric pathogens. Contact with animals is an important factor in *Cryptosporidium* infection in children, particularly in rural areas. In Ismailia Governorate, Egypt, Shoukry et al. reported that the highest *Cryptosporidium* infection rate was among children less than two months, and that 88% of infected children were in contact with animals compared with 12% of non-infected children (12). The authors also found that more than 20% of fecal samples examined from goats, cows, buffaloes, and sheep were positive for cryptosporidia. In Iraq, Al-Dabbagh et al. reported similar findings (24). In the past decade, the organisms have been reported in a wide range of domestic animals including camels, rabbits, turkeys, and quails with a prevalence rate ranging between <1% and 87% (mean = 15%). Also *Cryptosporidium* have been detected in stool samples from stray dogs in Egypt (25). Prevalence of *Cryptosporidium* in animals in several Arab countries is shown in Supplemental table 3.

### Contaminated drinking water and vegetables

Contaminated drinking water and vegetables appear to be an important source of *Cryptosporidium* infections in Arab countries. Shoukry et al. detected *Cryptosporidium* in 9%, 4%, 0.0%, and 7% of 75 samples each of tank water, canal water, bottled water, and underground water, respectively (12). El-Shazly et al. found *Cryptosporidium* in 3% of 840 potable water samples from seven districts in Dakahlia Governorate in Egypt (17). Hussein reported an outbreak that involved several individuals admitted to a local hospital in Nablus, Palestine, with symptoms of diarrhea, strong abdominal pain, and periodic vomiting (26). Stool samples from 30 patients involved in the outbreak were positive for *Cryptosporidium* spp. by microscopy and molecular methods. Using PCR-restriction fragment length polymorphism (RFLP) technique, they identified the parasite as *C. parvum*. The outbreak was traced to a local spring that was found to be contaminated with *C. parvum* by microscopy and PCR-RFLP. The parasite was not detected in all other drinking water sources in the city.

Al-Binali et al. in southwestern Saudi Arabia examined samples of leafy vegetables for intestinal parasites. They reported *Cryptosporidium* species in 8.3% (3/36) of watercress, and 2.8% (1/36) of leeks (27). They also reported that washing vegetables with tap water does little to remove parasites, including *Cryptosporidium* species, from vegetables. Unfortunately, the use of untreated sewage for crop irrigation is common in Arabic countries, and this may contribute to the high prevalence of cryptosporiadae reported for the region.

## Molecular studies

In the past decade, few studies reported molecular characterization of *Cryptosporidium* from immunocompetent and immunocompromised patients in the Arab world. Al-Brikan et al. examined 35 *Cryptosporidium*-positive fecal samples (by microscopy and ELISA) from children in Jeddah, Saudi Arabia, using molecular techniques (28). Of the samples, Al-Brikan et al. identified *C. hominis* in 13 (37%), *C. parvum* in 15 (42.9%), *C. meleagridis* in one (2.9%), *C. muris* in one (2.9%), and a mixed infection of *C. hominis* and *C. parvum* in one (2.9%). Essid et al., analyzing the eight *Cryptosporidium*-positive cases of immunocompromised children by PCR-RFLP to determine the species, showed that four were *C. hominis*, three were *C. parvum*, and one was *C. meleagridis* (6).

Applying molecular procedures, PCR-RFLP, and a 60-kDa glycoprotein (GP60)-based PCR sequencing tool, *C. hominis* and *C. parvum* are typed into subtype families designated as Ia, Ib, Id, Ie, and so forth for *C. hominis*, and as IIa, IIb, IIc, IId, and so forth for *C. parvum* (29). Subtyping methods are important in investigating *C. hominis* transmission in humans and *C. parvum* in humans and animals (30). Recently, Iqbal et al. determined the species/genotypes and subtype families of 83 *Cryptosporidium*-positive specimens from diarrheic Kuwaiti children by PCR-RFLP (31). They identified *C. parvum* in 73.5% (61/83) and *C. hominis* in 26.5% (22/83). Subtyping of *C. parvum* isolates showed the predominance of subtypes IIa and IId (80%), followed by IIc. The majority *C. hominis* isolates belonged to subtype Id (54.5%), followed by Ia (36.4%) and Ie.

## Treatment and prevention

Nitazoxanide is an antiprotozoal drug that works by stopping the growth of certain protozoa associated with diarrhea and is used to treat diarrhea caused by *Cryptosporidium* or *Giardia* in children and adults (32). In 2002, the US Food and Drug Administration (FDA) licensed nitazoxanide for the treatment of cryptosporidiosis in children 1 to 11 and, in 2004, for older children and adults. Recently, a placebo-controlled study carried out in the Nile Delta region of Egypt found that a 3-day course of nitazoxanide is effective in treating diarrhea and enteritis caused by *Cryptosporidium* species in non-immunodeficient patients 12 years of age or older (33). Nitazoxanide is also effective against *Giardia intestinalis* as well as other intestinal parasites. In a clinical setting where a definite diagnosis is not feasible or practical, nitazoxanide activity may provide a significant advantage against *Cryptosporidium* species, *G. intestinalis*, and other enteric parasites (34).

*Cryptosporidium* is resistant to common water treatment by chlorination. However, a water treatment plant's particle removal process removes more than 97% of *Cryptosporidium* oocysts (35). Although the use of treated water does not pose a serious health risk to immunocompetent individuals, immunocompromised persons are at higher risk of *Cryptosporidium* infection from using such waters, particularly for drinking. Currently available methods that completely eliminate *Cryptosporidium* in drinking water include the following: boiling of water, passing the water through a filter with pores smaller than *Cryptosporidium* (one micron or smaller), or using reverse osmosis filters. These methods can be used by individuals or for small groups of patients (e.g. in hospital units caring for immunocompromised patients). In addition, bottled water that is distilled, reverse osmosis treated, or filtered through a one-micron or smaller filter is another alternative.

## Conclusion

*Cryptosporidium* species, mainly *C. parvum*, are important causes of diarrhea in immunocompetent and immunocompromised persons, particularly children, in Arab countries. Contact with animals and contaminated waters are the main modes of transmission of cryptosporidia. Reports of *C. hominis* from the region indicate that person-to-person transmission is also important. Improving water treatment processes, protecting treated waters from contamination, and banning the use of untreated sewage for crop irrigation are priorities that should be addressed seriously in Arab countries. In addition, educational programs that promote personal, household, as well as food hygiene should be implemented by the health and environmental authorities in each Arab country. Cooperation between such countries in addressing these issues is of added benefit to the public. In the future, more studies employing molecular testing methods are needed to provide data on circulating species/genospecies and subtypes and their modes of transmission in the community. Finally, the availability of nitazoxanide for treating *Cryptosporidium* as well as other enteric parasitic infections should encourage clinicians in Arab countries requesting *Cryptosporidium* testing, which in turn may lead to an increase in case reports.

## References

[1] O'Donoghue PJ (1995). Cryptosporidium and cryptosporidiosis in man and animals. Int J Parasitol.

[2] Anonymous (2008). Cryptosporidiosis. World Organization for Animal Health.

[3] Ramirez NE, Ward LA, Sreevatsan S (2004). A review of the biology and epidemiology of cryptosporidiosis in humans and animals. Microbes Infect.

[4] Xiao L, Feng Y (2008). Zoonotic cryptosporidiosis. FEMS Immunol Med Microbiol.

[5] Abdel-Messih IA, Wierzba TF, Abu-Elyazeed R, Ibrahim AF, Ahmed SF, Kamal K (2005). Diarrhoea associated with *Cryptosporidium parvum* among young children of the Nile River Delta in Egypt. J Trop Pediatr.

[6] El-Mohamady H, Abdel-Messih IA, Youssef FG, Said M, Farag H, Shaheen HI (2006). Enteric pathogens associated with diarrhea in children in Fayoum, Egypt. Diagn Microbiol Infect Dis.

[7] Essid R, Mousli M, Aoun K, Abdelmalek R, Mellouli F, Kanoun F (2008). Identification of *Cryptosporidium* species infecting humans in Tunisia. Am J Trop Med Hyg.

[8] Nimri LF, Meqdam M (2004). Enteropathogens associated with cases of gastroenteritis in a rural population in Jordan. Clin Microbiol Infect.

[9] Garcia L (2001). Diagnostic medical parasitology.

[10] Helmy MM, Rashed LA, el-Garhy MF (2004). Molecular characterization of *Cryptosporidium parvum* isolates obtained from humans. J Egypt Soc Parasitol.

[11] Nimri LF, Elnasser Z, Batchoun R (2004). Polymicrobial infections in children with diarrhoea in a rural area of Jordan. FEMS Immunol Med Microbiol.

[12] Shoukry NM, Dawoud HA, Haridy FM (2009). Studies on zoonotic cryptosporidiosis parvum in Ismailia Governorate, Egypt. J Egypt Soc Parasitol.

[13] Al-Braiken FA, Amin A, Beeching NJ, Hommel H, Hart CA (2003). Detection of *Cryptosporidium* amongst diarrhoeic and asymptomatic children in Jeddah, Saudi Arabia. Ann Trop Med Parasitol.

[14] Al-Harthi SA (2004). Prevalence of intestinal parasites in schoolchildren in Makkah, Saudi Arabia. New Egypt J Med.

[15] Ben Ali M, Ghenghesh KS, Ben Aissa R, Abuhelfaia A, Dufani MA (2005). Etiology of childhood diarrhea in Zliten-Libya. Saudi Med J.

[16] El Shazly AM, Soltan DM, El-Sheikha HM, Sadek GS, Morsy AT (2007). Correlation of ELISA copro-antigen and oocysts count to the severity of cryptosporidiosis parvum in children. J Egypt Soc Parasitol.

[17] Mahgoub ES, Almahbashi A, Abdulatif B (2004). Cryptosporidiosis in children in a north Jordanian paediatric hospital. East Mediterr Health J.

[18] Rahouma A, Klena JD, Krema Z, Abobker AA, Treesh K, Franka E (2011). Enteric pathogens associated with childhood diarrhea in Tripoli-Libya. Am J Trop Med Hyg.

[19] Abu-Alrub SA, Abusada GM, Farraj MA, Essawi TA (2008). Prevalence of *Cryptosporidium spp*. in children with diarrhoea in the West Bank, Palestine. J Infect Developing Countries.

[20] Sanad MM, Al-Malki JS (2007). Cryptosporidiosis among immunocompromised patients in Saudi Arabia. J Egypt Soc Parasitol.

[21] Fathallah A, Saghrouni F, Madani B, Ben Rejeb N, Ben Said M (2004). Infantile cryptosporidiosis in Sousse area, Tunisia. Arch Pédiatr.

[22] Ben Abda I, Essid R, Mellouli F, Aoun K, Bejaoui M, Bouratbine A (2011). *Cryptosporidium* infection in patients with major histocompatibility complex class II deficiency syndrome in Tunisia: description of five cases (in French). Arch Pédiatr.

[23] Al-Madani AA, Mahfouz AA (1995). Prevalence of intestinal parasitic infections among Asian female house keepers in Abha district, Saudi Arabia. Southeast Asian J Trop Med Pub Health.

[24] Al-Dabbagh NY, Abbassa ET, Ahmed MM (2010). Evaluation of risk factors of cryptosporidiosis in children: a case-control study in Mosul city. Tikrit Med J.

[25] El-Madawy RS, Khalifa NO, Khater HF (2010). Detection of cryptosporidial infection among Egyptian stray dogs by using *Cryptosporidium parvum* outer wall protein gene. Bulg J Vet Med.

[26] Hussein AS (2011). *Cryptosporidium parvum* causes gastroenteritis epidemics in the Nablus region of Palestine. Trop Med Intern Health.

[27] Al-Binali AM, Bello CS, El-Shewy K, Abdulla SE (2006). The prevalence of parasites in commonly used leafy vegetables in South Western Saudi Arabia. Saudi Med J.

[28] Al-Brikan FA, Salem HS, Beeching N, Hilal N (2008). Multilocus genetic analysis of *Cryptosporidium* isolates from Saudi Arabia. J Egypt Soc Parasitol.

[29] Sulaiman IM, Hira PR, Zhou L, Al-Ali FM, Al-Shelahi FA, Shweiki HM (2005). Unique endemicity of cryptosporidiosis in children in Kuwait. J Clin Microbiol.

[30] Xiao L (2010). Molecular epidemiology of cryptosporidiosis: an update. Experimental Parasitol.

[31] Iqbal J, Khalid N, Hira PR (2011). Cryptosporidiosis in Kuwaiti children: association of clinical characteristics with *Cryptosporidium* species and subtypes. J Med Microbiol.

[32] MedlinePlus Nitazoxanide. http://www.nlm.nih.gov/medlineplus/druginfo/meds/a603017.html.

[33] Rossignol J-F, Kabil SM, El-Gohary Y, Younis AM (2006). Effect of Nitazoxanide in diarrhea and enteritis caused by *Cryptosporidium* species. Clin Gastroenterol Hepatol.

[34] Rossignol J-F (2010). Cryptosporidium and Giardia: Treatment options and prospects for new drugs. Experimental Parasitol.

[35] Roanoke Rapids Sanitary District Cryptosporidium. http://rrsd.org/crypto.htm.

